# Stiffer, Stronger and Centrosymmetrical Class of Pentamodal Mechanical Metamaterials

**DOI:** 10.3390/ma12213470

**Published:** 2019-10-23

**Authors:** Yan Huang, Xiaozhe Zhang, Muamer Kadic, Gongying Liang

**Affiliations:** 1 School of Science, Xi’an Technological University, Xi’ an 710021, China; 2 School of Materials Science and Engineering, Xi’an Polytechnic University, Xi’an 710048, China; zhangxiaozhe0303@126.com; 3 Institut FEMTO-ST, UMR 6174, CNRS, Université de Bourgogne Franche-Comté, 25000 Besançon, France; muamer.kadic@kit.edu; 4 School of Science, Xi’ an Jiaotong University, Xi’an 710049, China; gyliang@mail.xjtu.edu.cn

**Keywords:** pentamode, mechanical metamaterial, phonon band structure, centrosymmetrical

## Abstract

Pentamode metamaterials have been used as a crucial element to achieve elastical unfeelability cloaking devices. They are seen as potentially fragile and not simple for integration in anisotropic structures due to a non-centrosymmetric crystalline structure. Here, we introduce a new class of pentamode metamaterial with centrosymmetry, which shows better performances regarding stiffness, toughness, stability and size dependence. The phonon band structure is calculated based on the finite element method, and the pentamodal properties are evaluated by analyzing the single band gap and the ratio of bulk and shear modulus. The Poisson’s ratio becomes isotropic and close to 0.5 in the limit of small double-cone connections. Stability and scalability analysis results show that the critical load factor of this structure is obviously higher than the classical pentamode structure under the same static elastic properties, and the Young’s modulus gradually converges to a stable value (the infinite case) with an increasing number of unit cells.

## 1. Introduction

Metamaterials are rationally designed composite structures made of building blocks (or unit cells or even meta-atoms), which are composed of one or more constituent bulk materials. They are said to have effective properties beyond the standard material [1,2].

For example, in optics, they have been introduced theoretically by Veselago [3] as the necessary material for negative refraction of light. Later in 2000, Pendry proposed how to achieve such negative refractive index slabs using a thin metal layer and to get a perfect lens [4]. Interestingly, many scientists focused on trying to design such material but the general laws of physics (Kramer‒Kronig relations) tell us that such properties can only be found at a single frequency. In the simplest Drude‒Lorenz model, one can get a negative permittivity but one must pay the price by having a non-zero imaginary part. More generally, having materials that are really going beyond the bounds is very often simply impossible according to physics laws [1,2].

Now, let us look at the mechanical properties of material. They are often summarized in the so-called elasticity tensor [1,5,6]. One generally considers that any elasticity tensor must be positive definite. This constraint has been relaxed by the idea of convexity by Milton [7,8,9]. In the isotropic case, the elasticity tensor has only two different eigenvalues. The first is proportional to the bulk modulus of the material and the second, five times degenerated, to the shear modulus. This means that when focusing on isotropic materials, one should only pay attention to these two moduli.

Milton and Cherkaev [10] in parallel with Sigmund [11] asked the following question in 1995: Which elasticity tensors are realizable? Can one for example cancel all shear related eigenvalues of an elastic tensor? Sigmund [11] used topological optimization to find a structure corresponding to such material with prescribed constitutive property, i.e., the Poisson’s ratio closes to 0.5. Milton and Cherkaev [10] have proposed a structure based on intuition, group of symmetry and idealized joins. They firstly proposed such a structure as the “pentamode” from mathematical analysis. This pentamode structure is composed of double cones, and the joint points form a diamond lattice. Later, the mechanical property of such pentamode structure was realized and thoroughly studied [12,13,14,15,16,17,18,19,20,21,22,23]. Kadic et al. [12] fabricated the polymer pentamode structure using state-of-the-art dip-in direct-laser-writing (DLW) optical lithography. Then, they took advantage of the scalability of continuum mechanics and fabricated a much larger macroscopic version of the pentamode structure to directly perform elastic measurements [14]. Meanwhile, they also performed a series of numerical simulation calculations, including phonon band structure, elastic mechanic property and so on, to obtain a comprehensive analysis of the pentamode structure from theory and experiments [13,15,16]. In addition, Amendola et al. [17] experimentally investigated the mechanical response of an additively manufactured metallic pentamode structure confined between stiffening plates, and also conducted finite element simulations to study the bending dominated response of layered mechanic metamaterials alternating such pentamode lattices and confinement plates [18]. The special mechanical property of pentamode material, i.e., difficult to compress and easy to deform, shows promising application in meta-liquids, shear wave band-gap systems [19,20], and innovative seismic isolation devices [17]. 

The pentamode structure has even also been used for practical applications, such as elastic unfeelability [24]. The core-shell elasto-mechanical cloak shows good cloaking performance under uniaxial pushing conditions and conceals the obstacle. Therefore, the development of pentamode mechanical metamaterials will enable three-dimensional transformation elastodynamic architectures and significantly improve our ability to steer waves and energy fluxes in mechanics.

Up to the present, most pentamode metamaterials are based on Milton’s diamond-like pentamode structure. However, in the process of investigation, this structure is not very practical to fabricate nor to be used due to two main arguments: (i) fragility due to small connections and (ii) sensibility to scaling due to also the non-centrosymmetry. Here, in this paper we propose a new class of pentamodal metamaterials with centrosymmetry that show better stability, better performances (stiffness and toughness) and for which effective properties depend much less on the number of unit cells than the previous proposals [12,13,14,15,16,17,18,19,20,21,22,25,26,27].

## 2. Modeling and Method 

**Figure 1:**
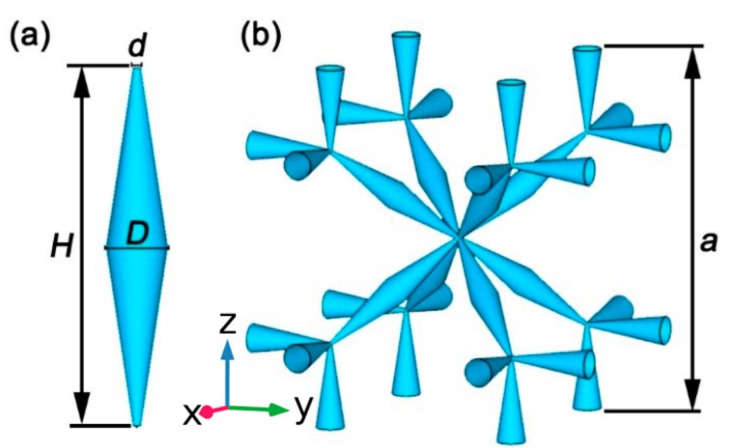
Model and unit cell of the pentamode structure. (**a**) Basic building element of a double-cone. (**b**) Model of pentamode structure in a simple cubic lattice.

In order to design a pentamodal structure, the key element is a tetragonal element composed of four double cones connected in a small point-like join. Then, we must carefully consider how we connect these primitive blocks to assemble a unit cell. Here, we emphasize that a missing but important aspect is the centrosymmetry, and we propose the unit cell shown in Figure 1.

Taking a closer look, pairs of cones are connected at their thick ends with a diameter D to form a complete double-cone unit, just as shown in Figure 1a. The double-cone elements then make contact with each other at their thin ends with a diameter d, and these connection points form a simple cubic lattice. The relation between the lattice constant a and the length of the double-cone *H* is $H=\left(2\sqrt{3}-3\right)a$. By periodically repeating the unit cell shown in Figure 1b, the complete pentamode structure can be obtained. 

In this paper, based on the finite-element method, we performed a series of numerical simulation calculations using COMSOL Multiphysics (Version 5.3, COMSOL, France). Specifically speaking, we firstly numerically calculated the phonon band structure of this model in the structural mechanics module, based on the Bloch theorem and by solving the elasto-dynamic equation. Then, combining the generalized Hook’s law and the elastic wave equations, we can retrieve the elasticity tensor and then the mechanic modulus from the expressions [28]:

(1) $$C_{44}=\rho {\left(v_{110}^{T,z}\right)}^{2}$$

(2) $$C_{12}=\rho {\left(v_{110}^{L}\right)}^{2}-C_{44}-\rho {\left(v_{110}^{T,xy}\right)}^{2}$$

(3) $$C_{11}=2\rho {\left(v_{110}^{T,xy}\right)}^{2}+C_{12}$$

(4) $$G=C_{44}$$

(5) $$B=\left(C_{11}+2C_{12}\right)/3$$

where ρ represents the mass density of the pentamode structure, which is given by the volume filling fraction f times the mass density $\rho_{0}$ of the constituent material, i.e., $\rho =f\rho_{0}$ [16,29,30,31,32,33]. $C_{11},\;C_{12}$ and $C_{44}$ denote the three independent elastic constants of the cubic lattice. Based on the above parameters and the expressions, we further calculated the Poisson’s ratio of the pentamode structure, and finally, we performed stationary analysis in the structural mechanical module to study the stability of the structure, and also, static calculations were performed for the finite metamaterial samples containing *N* × *N* × 2*N* extended unit cells (see Figure 1b) to verify the scalability.

## 3. Results and Discussion

In order to quantify the performances of the new structure we first computed the dispersion relation. The constituent material of the pentamode structure we consider here is a polymer with mass density $\rho_{0}=1190\;kg/m^{3}$, Poisson’s ratio ν=0.4 and Young’s modulus E = 3 GPa.

**Figure 2:**
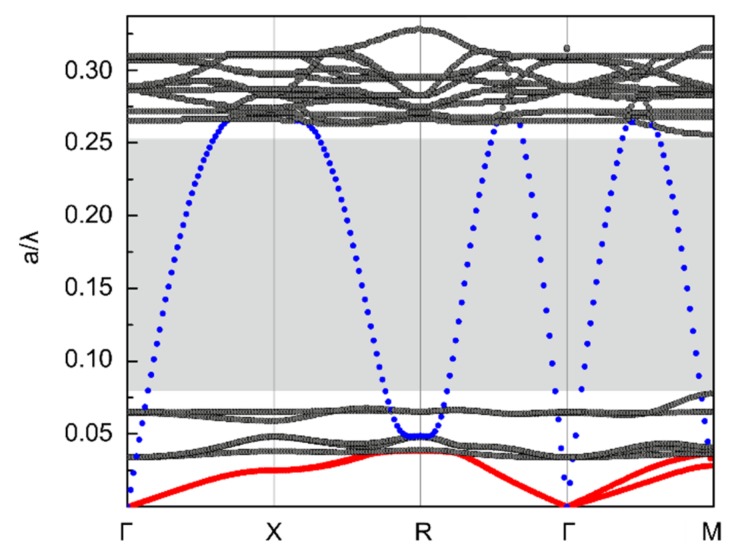
Phonon band structure of the pentamode structure (with geometrical parameters D/a = 2/37.3 and d/a = 0.5/37.3). The first two branches emphasized using red dots and the third using blue dots represent, respectively, are two transverse wave modes and a longitudinal wave mode. The light grey region is the single-mode band gap, in which only the longitudinal wave can propagate.

The phonon band structure was calculated with COMSOL Multiphysics using the structural mechanics module. The results of the pentamode structure are shown in Figure 2. To make clear the vibration modes of the dispersion relation curves, the different modes are colored differently (see caption of Figure 2).

The branches 1 and 2 (depicted in red) correspond to the first elastic transverse modes. Similarly, the branch 3 (depicted in blue) corresponds to the longitudinal mode. It becomes immediately clear that the light grey region in the phonon band structure is the single-mode band gap, in which the transverse waves are inhibited and only the longitudinal wave can propagate.

By extracting the slope data of acoustic branches in ΓM direction, the phase velocities of transverse waves and longitudinal wave were obtained. Then, the bulk modulus B and shear modulus G were deduced from the expressions (1–5). Finally, the calculated B/G ratio of the pentamode structure was as large as 311. Therefore, by combining the higher B/G ratio than traditional materials and the existence of the single-mode band gap, we could confirm the pentamodal property of the new structure from the aspect of physical property. Furthermore, from the aspect of mathematical analysis, five of the six diagonal elements of the diagonalized 6 × 6 elasticity tensor of the pentamode material were zero, and only one was non-zero. This means that it can only support a single stress, and it satisfies the mathematical definition of the “pentamode”.

**Figure 3:**
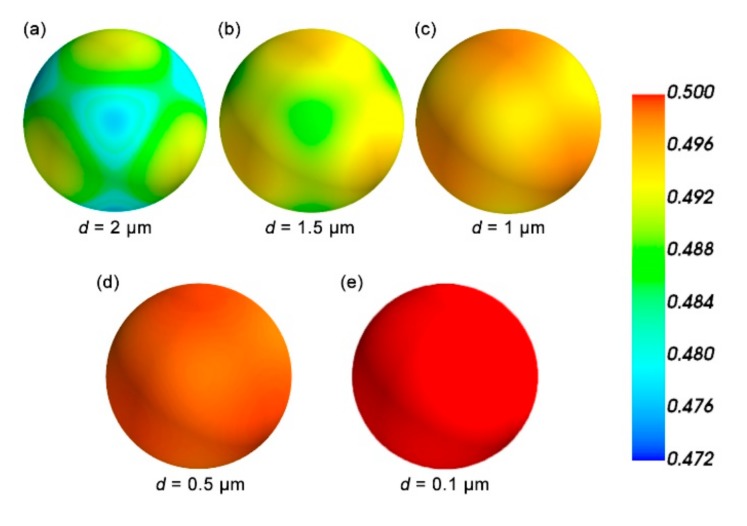
Three-dimensional polar diagram of the effective Poisson’s ratio ν(a = 37.3 μm, D = 2 μm). The length of the vector from the origin to the surface is proportional to the modulus of Poisson’s ratio. The small diameter d has been used as parameter: (**a**) d = 2 μm, (**b**) d = 1.5 μm, (**c**) d = 1 μm, (**d**) d = 0.5 μm and (**e**) d = 0.1 μm.

Then, to check the isotropy of the new structure, we plotted the direction dependence of the Poisson’s matrix ν of the pentamode structure with the periodic boundary conditions imposed on the unit cell in Figure 3. The Poisson’s ratio (matrix here) ν is proportional to the length of the vector from the origin to the depicted surface. In general, the Poisson’s ratio of the ideal pentamode approaches 0.5, and the Poisson’s ratio of this structure, as shown in Figure 3, is in the range from 0.472 to 0.5. What is more, the difference of Poisson’s ratio for different directions is minimal. The result shows that ν becomes more and more isotropic when d decreases, leading to the ultimate limit of 0.5 (ideal pentamode).

**Figure 4:**
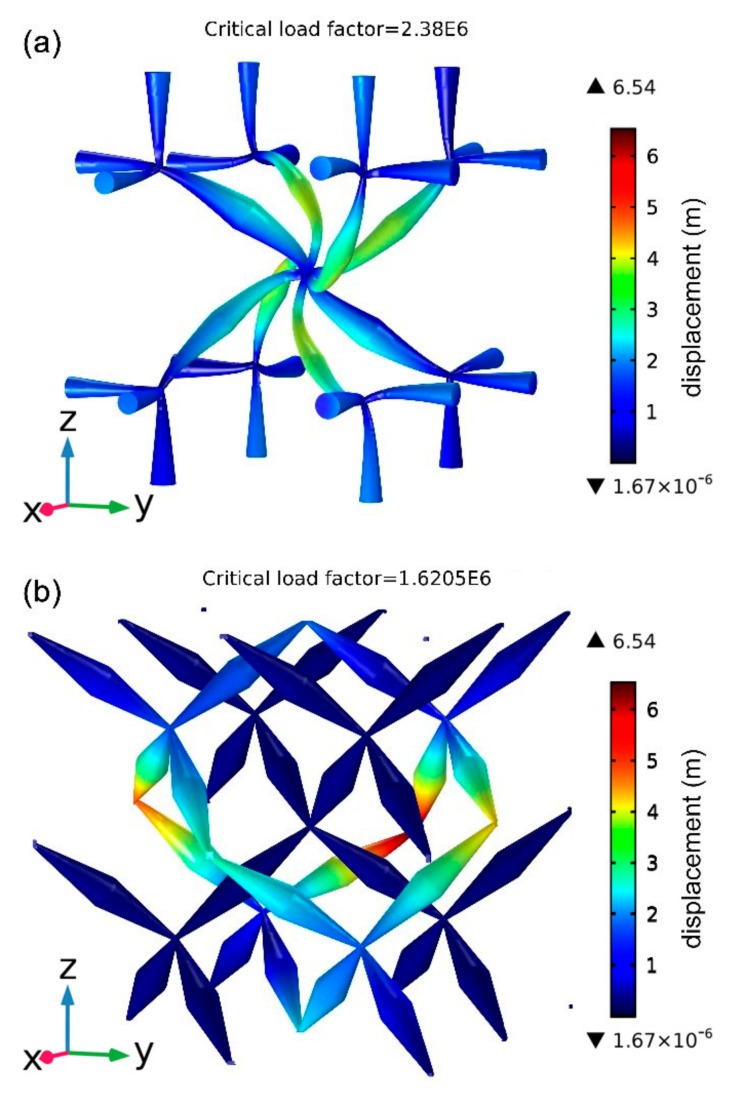
Stability comparison between (**a**) the new pentamode structure (a = 37.3 μm, D = 2 μm and d = 0.5 μm) in this paper and (**b**) the classic pentamode structure (a = 37.3 μm, D = 3.05 μm and d = 0.43 μm). The critical load factors are (**a**) 2.38 × 10^6^ and (**b**) 1.62 × 10^6^, respectively.

Following this, we performed the stability analyses for the pentamode structure. To enable direct comparison, we chose the new pentamode structure (a = 37.3 μm, D = 2 μm and d = 0.5 μm) in this paper and the classic pentamode structure (a = 37.3 μm, D = 3.05 μm and d = 0.43 μm) with diamond lattice studied by Kadic et al. [12] to compare. For the condition of the above geometrical parameters, the two structures have almost the same B and G. Specifically, $B_{1}=1.73\times 10^{6}\;\mathrm{Pa}$, $G_{1}=5.57\times 10^{3}\;\mathrm{Pa}$ for the new pentamode structure in this paper, and $B_{2}=1.90\times 10^{6}\;\mathrm{Pa}$, $G_{2}=5.53\times 10^{3}\;\mathrm{Pa}$ for the classic pentamode structure. We simultaneously applied the equal and opposite force on the top and bottom face, the front and back face, and the left and right face of the two structures to compress them. By exerting load on the two structures, the calculated critical load factors are $2.38\times 10^{6}$ for new the pentamode structure and $1.62\times 10^{6}$ for the classic pentamode structure, respectively, as shown in Figure 4. This means that the new pentamode structure can support a bigger load than the classic structure, and thus it should be more stable.

**Figure 5:**
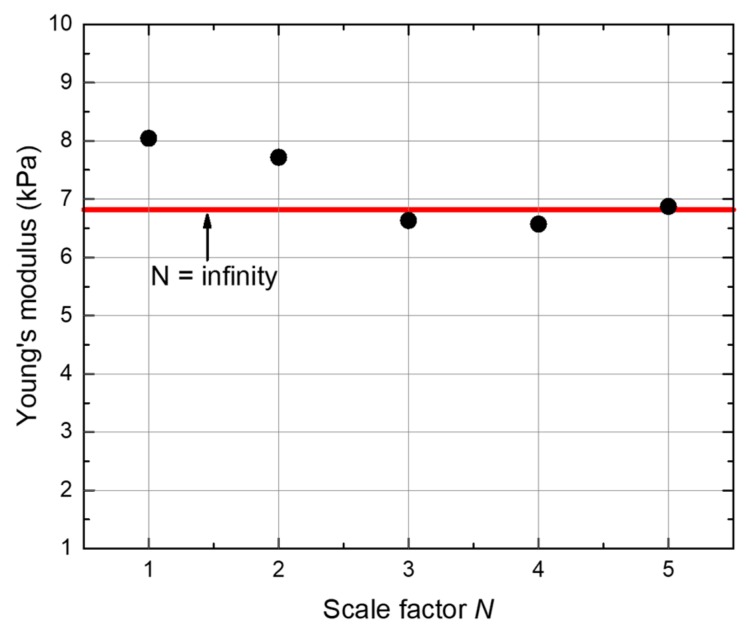
Young’s moduli of the pentamode structure (a = 37.3 μm, D = 2 μm and d = 0.5 μm) with *N* × *N* × 2*N* extended unit cells (*N* = 1, 2, 3, 4, 5). The horizontal line refers to the value of Young’s modulus for the infinite case.

Finally, we performed a simple compressional test based on Young’s principle by performing compressional experiments on different sample sizes composed of the same unit cells. The compression experiment samples included *N* × *N* × 2*N* extended unit cells, and *N* = 1, 2, 3, 4, 5. The simulation details and boundary conditions were as follows: the bottom face of the sample is fixed, and a constant force is applied on the top face along the z-direction. This means that we applied the normal stress on the top face and compressed the sample along the negative z-direction. Then, we could obtain the displacement of the top face of the sample after solving. Finally, the Young’s modulus of the sample could be derived based on its definition, i.e., the ratio of longitudinal stress and longitudinal strain, as is shown in Figure 5. Results show that the Young’s modulus of this pentamode structure gradually converges to 6.82 kPa (the infinite case) for increasing *N* from 1 to 5, and this phenomenon indicates that the pentamode structure shows good performance on scalability. In other words, it is not necessary to have too many unit cells in order to mimic an effective medium close to the infinite case [2,34,35,36]. 

## 4. Conclusions

In conclusion, we have shown a new class of pentamodal metamaterial with better elastic properties and a higher stability and scalability. A ratio of B/G of more than 300 and complete single-band gap have been obtained. The Poisson’s ratio is nearly isotropic and approaches the limit of ideal pentamode metamaterial of 0.5 as the small internal connection d decreases. Five of the six eigenvalues of elasticity tensor are zero, and the pentamodal property of the new structure is characterized from the two aspects, i.e., the physical property and the mathematic definition. Moreover, under the same external force conditions, the new pentamode structure shows a twist in the center, which allows it to support a greater load than the previous structure. The Young’s modulus of the new structure converges to 6.82 kPa (the infinite case) for increasing *N* from 1 to 5, and one will not need to have too many unit cells in order to mimic an effective medium. Therefore, the higher stability and scalability makes it easily realizable in experiments, and it provides an available candidate for three-dimensional elastic cloaks in the future.
